# Body Adiposity, But Not Elements of Objectively Measured Sedentary Behavior or Physical Activity, Is Associated With Circulating Liver Enzymes in Adults With Overweight and Obesity

**DOI:** 10.3389/fendo.2021.655756

**Published:** 2021-04-20

**Authors:** Saara Laine, Tanja Sjöros, Henri Vähä-Ypyä, Taru Garthwaite, Eliisa Löyttyniemi, Harri Sievänen, Tommi Vasankari, Juhani Knuuti, Ilkka H. A. Heinonen

**Affiliations:** ^1^ Turku PET Centre, University of Turku and Turku University Hospital, Turku, Finland; ^2^ The UKK-Institute for Health Promotion Research, Tampere, Finland; ^3^ Department of Biostatistics, University of Turku, Turku, Finland; ^4^ Faculty of Medicine and Health Technology, Tampere University, Tampere, Finland; ^5^ Rydberg Laboratory of Applied Sciences, University of Halmstad, Halmstad, Sweden

**Keywords:** physical activity, obesity, adiposity, liver, liver enzymes, sedentary behavior

## Abstract

**Objective:**

We studied the associations between accelerometer-measured sedentary behavior (SB) and habitual physical activity (PA) as well as markers of body adiposity and other cardiometabolic risk factors with liver enzymes alanine aminotransferase (ALT), aspartate aminotransferase (AST) and γ-glutamyltransferase (GGT).

**Methods:**

A total of 144 middle-aged adults (mean age 57 (SD 6.5) years) with overweight or obesity (mean body mass index [BMI] 31.8 [SD 3.9] kg/m^2^) participated. Different components of SB (sitting, lying) and PA (standing, breaks in SB, daily steps, light PA, moderate-to-vigorous PA and total PA) were measured with validated hip-worn accelerometers for four consecutive weeks (mean 25 days, [SD 4]). Fasting venous blood samples were analysed using standard assays. The associations were examined with Pearson’s partial correlation coefficient test and linear mixed model.

**Results:**

Among 102 women and 42 men accelerometer measured SB or the elements of PA were not associated with circulating liver enzymes. When adjusted for age and sex, liver enzymes correlated positively with BMI and waist circumference (WC) (ALT r=0.34, p<0.0001, r=0.41, < 0.0001, AST r=0.17, p=0.049, r=0.26, p=0.002, GGT r=0.29, p=0.0005, r=0.32, p < 0.0001, respectively). SB proportion associated positively with BMI (r=0.21, p=0.008) and WC (r=0.27, p=0.001). Components of PA associated negatively with BMI (MVPA r=-0.23, p=0.005, daily steps r=-0.30, p<0.0001 and breaks in sedentary time r=-0.32, p<0.0001), as well as with WC (breaks in SB r=-0.35, p<0.0001, MVPA r=-0.26, p=0.002, daily steps r=-0.31, p<0.0001, standing time r=-0.27, p=0.001). Liver enzymes associated positively with common cardiometabolic markers such as resting heart rate (ALT; β=0.17, p=0.03, AST; β=0.25, p=0.002, GGT; β=0.23, p=0.004) and systolic/diastolic blood pressure (ALT β=0.20, p=0.01, β=0.22, p=0.005, AST (only diastolic) β=0.23, p=0.006, GGT β=0.19, p=0.02, = 0.23, p=0.004, respectively), fasting insulin (ALT β=0.41, p<0.0001, AST β=0.36, p=0.0003, GGT β=0.20, p=0.04) and insulin resistance index (ALT β=0.42, p<0.0001, AST β=0.36, p=0.0003, GGT β=0.21, p=0.03), even after adjustment with BMI.

**Conclusions:**

Liver enzymes correlate with body adiposity and appear to cluster with other common cardiometabolic risk factors, even independently of body adiposity. SB and PA appear not to be essential in modulating the levels of circulating liver enzymes.

## Introduction

Alanine aminotransferase (ALT), aspartate aminotransferase (AST) and γ-glutamyltransferase (GGT) are enzymes found in the plasma and other tissues but are most common in the liver (1). These enzymes are generally considered biomarkers for hepatocellular injury (2), and their increased concentration in the plasma indicates that the liver is damaged. Excess accumulation of fat in the liver due to increased body adiposity (3) is thus one plausible cause for the increased levels of these enzymes in the blood (4, 5). Obesity has been associated with elevated levels of circulating liver enzymes (6, 7), which may increase the risk of developing metabolic diseases such as non-alcoholic fatty liver disease (NAFLD) (4, 8).

Leisure time physical activity (PA) is known to be associated with low body mass index (BMI) while increased amount of sitting is positively associated with BMI, even after adjustments for multiple common confounders including dietary factors and genetic predisposition (9–11). Furthermore, self-reported TV viewing a proxy of sedentary behavior (SB) (12) associates positively with markers of fatty liver risk, although adjustment for BMI attenuates this association (13). Previous studies concerning the associations between liver enzymes and objectively measured PA in people with obesity or overweight are limited. Studies have mainly focused on adolescents (14–16) and in the few existing studies in adults the device tracking time has been short; only 4-7 days (17, 18).

Consequently, the aim of this study was to examine the associations between specific liver enzymes and elements of SB (lying, sitting, total sedentary time) and PA (breaks in sedentary time, standing, daily steps, light physical activity [LPA], moderate-to-vigorous PA [MVPA] and total PA [LPA and MVPA together] in sedentary inactive adults with overweight or obesity. Additionally, our aim was to examine the associations between liver enzymes and markers of body adiposity (BMI and waist circumference [WC]) and other cardiometabolic markers (e.g., blood pressure, insulin resistance and resting heart rate [HR]).

We hypothesized that liver enzymes are positively associated with BMI, WC, blood pressure, insulin resistance, resting heart rate and with SB, whereas components of PA (LPA and MVPA) show at least modest negative associations. We further hypothesized that liver enzymes show a positive association with sitting time and a negative association with standing and especially breaks in SB, and that these associations remain significant after adjustments for common confounding factors such as age, sex and BMI.

## Methods

This study used cross-sectional data from the screening phase of an intervention study (Medical and physiological benefits of reduced sitting), registered at Clinicaltrials.gov (NCT03101228). The study was performed at the Turku PET Centre, Turku, Finland between April 2017 and May 2019. The study was approved by the Ethics Committee of the Hospital District of Southwestern Finland (Turku, Finland, study permission no TO5/026/17) and was carried out according to good clinical practice and the Declaration of Helsinki.

### Participants

The participants were recruited from the local community by newspaper advertisements and bulletin leaflets as previously reported (19). Selection criteria (for this study and the intervention study (NCT03101228)) were the following: middle-aged (40-65 years), overweight or obesity (BMI 25-40 kg/m^2^), and self-reported insufficient PA (less than 120 minutes of moderate intensity exercise per week), and high sedentary time (sitting a major proportion (≥ 10 hours) of the day at work and/or leisure time). The exclusion criteria were the following: history of a cardiac event, insulin or medically treated diabetes, abundant use of alcohol, use of narcotics, smoking of tobacco or consuming snuff tobacco, and any chronic disease or condition that could create a hazard to the subject safety or endanger the study procedures.

Alcohol consumption was determined by a questionnaire as units/week. One unit contains about 10 to 14 grams of alcohol. Abundant use was considered consumption higher than the national limits for high risk in Finland (more than 12 units for women and 23 units for men).

### SB and PA Measurements

SB and PA were measured for four weeks with hip-worn tri-axial accelerometers (UKK AM30, UKK-Institute, Tampere, Finland) as previously reported (19). Participants were instructed to wear the accelerometer during waking hours, except for activities where the devise would be exposed to water.

The collected accelerometer data was analyzed in six-second epochs using validated mean amplitude deviation (MAD) method (20). LPA was defined as 1.5-2.9 METs (MAD 22.5-91.5 m*g*), and MVPA as ≥ 3.0 METs (MAD > 91.5 m*g*). MAD values were further converted to metabolic equivalents (METs). Additionally, proportions of different activity intensities (PA, LPA and MVPA) per day were calculated, and presented as percentage of wear time. Total PA was calculated by adding LPA to MVPA. Number of participants that gained any vigorous PA was very low and the duration of such activity was very short (only few minutes). Therefore, moderate PA and vigorous PA values were added together and presented as MVPA.

Body posture was determined with angle for posture estimation (APE) method only for the epochs when the estimated MET value was lower than the commonly considered 1.5 MET cut point for SB (MAD less than 22.5 m*g)* (21). The epochs having APE values less than 11.6° were classified as standing and epochs having APE value at least 11.6° as SB, including sitting and lying. The APE value for separating sitting from lying was 73° (21). In the reference (21) the optimal cut-off point for separating sitting from lying is 64.9 degrees. However, in the reference the smallest measured APE value for lying is 73.9 degrees and highest APE value for sitting 55.9 degrees. We decided to use 73 degrees as a cut-off point. Thus, reclining is more likely classified as sitting. In addition to actual time (h/day) spent sedentary, standing, and in PA, proportions of accelerometer wear time in SB (SB %), standing (standing %) were calculated.

The number of breaks in sedentary time denoted the number of sedentary periods during which the one-minute exponential moving average of the MAD value was less than 22.5 m*g* and which ended-up with a clear vertical acceleration and subsequent standing position or movement (21).

The step detection algorithm splits the measured acceleration into vertical and horizontal components. The vertical component is band-pass filtered (1 – 4 Hz) and positive values are integrated. When the integral value exceeds the specified limit, a step is detected. The step algorithm requires about 3 km/h walking speed to detect every step (21).

A period was classified as a non-wear time, if the raw acceleration of each three-measurement axis remained within 187.5 mg range for at least for 30 min time. For a valid data collection, wear time of 10-19 h/day on at least four days was required.

### Liver Enzymes and Other Cardiometabolic Markers in Plasma

Venous blood samples were drawn after at least 10 h of fasting and analyzed at the Turku University Hospital Laboratory. ALT and AST were determined by photometric (IFCC) method (Cobas 8000 c702 Analyzer, Roche Diagnostics GmbH, Mannheim, Germany) (P-AST was analyzed until 27.2.2019 with Cobas 8000 c 502 Analyzer, Roche Diagnostics GmbH, Mannheim, Germany). GGT was determined with enzymatic colorimetric assay (Cobas 8000 c702 Analyzer, Roche Diagnostics GmbH, Mannheim, Germany).

Plasma insulin was determined by electrochemiluminescence immunoassay (Cobas 8000 e801, Roche Diagnostics GmbH, Mannheim, Germany). Plasma glucose was determined by enzymatic reference method with hexokinase GLUC3; and plasma triglycerides, total low-density lipoprotein (LDL) and total high-density lipoprotein (HDL) cholesterol by enzymatic colorimetric tests (Cobas 8000 c702, Roche Diagnostics GmbH, Mannheim, Germany). Hemoglobin A_1c_ (HbA_1c_) was determined by turbidimetric inhibition immunoassay (Cobas 6000 c501, Roche Diagnostics GmbH, Mannheim, Germany). Homeostatic model assessment of insulin resistance (HOMA-IR) was calculated using the formula: fasting glucose (mmol/l) x fasting insulin (µmol/l)/22.5.

### Anthropometrics, Blood Pressure and Resting Heart Rate

Body mass index (BMI), waist circumference (WC), and blood pressure were measured prior to starting the accelerometer measurements. Blood pressure and resting heart rate were measured using a digital blood pressure monitor (Apteq AE701f, Rossmax International LtD, Taipei, Taiwan) in a seated position after at least 10 min of sitting. The mean of 2-3 measurements was used as the outcome measure. Body weight was measured by scales (Seca 797, Vogel & Halke, Hamburg, Germany) in light clothing. Body height was measured barefooted with a wall-mounted stadiometer. BMI was calculated using measured weight (kg) and height (m) (BMI = kg/m^2^). WC was measured with a flexible measuring tape midline between the iliac crest and the lowest rib, and the measurement was repeated twice or until the same measure was obtained twice. The anthropometric variables were measured during the recruitment interview under standard conditions. The hour of the day was chosen by the participant according to convenience. All the measurements were performed by the same researcher to ensure standardized measurements.

### Statistical Methods

The associations between ALT, AST and GGT (dependent variables) and health markers, SB, and PA measures (independent variables) were examined with Pearson’s partial correlation coefficient test and linear mixed model. Unpaired t-test was used to detect differences between sexes. There was a significant difference in liver enzyme levels between men and women and therefore sex was included as a covariate in all analysis. In the linear model BMI was added to the model to adjust for confounding overweight and obesity; and for GTT (marker of alcohol use) related outcomes, the use of alcohol consumption was added to the model. Additionally, we added the accelerometer wear time to the model and we also ran following linear mixed model for outcomes (liver enzymes) = sex + age + and PA measure + age x PA + sex x PA. Normality of distribution was assessed by visual evaluation, Shapiro-Wilk test and with logarithmic transformations. Logarithmic (log10) transformations were performed to ALT, AST, GGT and HOMA-IR. Specific sample size was not determined for the present study as this dataset consist of screening phase of a clinical trial. Multicollinearity was controlled for with variance inflation factor in all the models. All the values were below five and thus considered not to have multicollinearity issues. Missing data was handled by pairwise deletion. Out of 144 participants, only four had missing values: fasting blood samples are missing for two participants as they failed to visit the laboratory and resting heart rate values are missing from two participants due to incomplete documentation. If not otherwise stated, data are expressed as mean and standard deviation (SD), standardized β coefficients and 95% CI of unstandardized **(**B) values. The level of statistical significance was set at 5% (two-tailed). All analyses were carried out with the JMP pro 13.1 for Windows (SAS Institute Inc., Cary, NC, USA) and IBM SPSS Statistics for Windows, Version 27.0 (IBM Corp, Armonk, NY, USA).

## Results

### Characteristics of the Participants

Participants’ baseline characteristics grouped by sex are presented in **Table 1**. Out of 263 screened participants, 102 women and 42 men fulfilled the inclusion criteria and completed the accelerometer measurements and were included in the analyses. The mean accelerometer wear time was 14.4 (SD 1.0) h/day, and the mean duration of the measurement was 25 (SD 4) days. Sixty-two % of the participants were obese (BMI > 30 kg/m^2^) and 38% were overweight (BMI 25.0 to < 30). Fifty % of the participants had medication for elevated blood pressure and 11% for elevated blood cholesterol. Some participants also reported use of hormonal medication (14.6%), thyroid medication (13.8%), antidepressants (13.2%), gastrointestinal medication (11.1%), pain medication (9.7%), rheumatoid or osteoarthritis medication (6.9%), allergy (5.6%) or asthma medication (3.5%), medication for urinary problems (4.2%), anticoagulants (4.2%), sleep medication (3.5%), medication for vision and hearing related issues (2.8%), migraine medication (2.1%), medication for restless legs syndrome (1.4%), psoriasis medication (0.7%) and epilepsy medication (0.7%).

**Table 1:** Characteristics of the study participants by sex. If not otherwise stated, the results are reported as mean (SD).

	Men	Women
n, (% of total)	42 (29)	102 (71)
Age, years	58.0 (6.0)	56.4 (6.7)
**Anthropometrics**		
BMI, kg/m^2^	31.8 (3.6)	31.7 (4.2)
Waist, cm	116.3 (11.0)***	106.7 (10.4)***
**Health measurements**		
ALT, U/l	37 (20) **	28 (14)**
AST, U/l	29 (10)	27 (7)
GGT, U/l	40 (19)**	33 (33)**
Alcohol consumption, units (10-14g of alcohol)/week	5 (6)**	2 (2)**
Systolic blood pressure, mmHg	149 (19)	147 (20)
Diastolic blood pressure, mmHg	91 (11)	90 (12)
Resting heart rate, bpm	70 (11)	71 (11)
Blood pressure medication, n (%)	23 (55)*	34 (33)*
Cholesterol medication, n (%)	8 (11)	11 (11)
f-Glucose, mmol/l	5.9 (0.7)	5.8 (0.9)
f-Insulin, µmol/l	16 (10)**	12 (7)**
HOMA-IR	4.2 (3.0)*	3.2 (2.4)*
Triglycerides, mmol/l	1.6 (0.9)	1.4 (0.8)
Cholesterol, mmol/l	5.0 (0.7)*	5.4 (0.9)*
HDL-cholesterol, mmol/l	1.3 (0.3)***	1.7 (0.4)***
LDL-cholesterol, mmol/l	3.4 (0.7)	3.5 (0.9)
HbA_1c_, mmol/mol	38 (5)	37 (6)
**Sedentary behavior**		
Lying time, h/days	2.0 (1.1)***	1.3 (0.7)***
Sitting time, h/day	8.1 (1.4)	8.1 (1.1)
Sedentary time, h/day	10.1 (1.2)**	9.4 (1.3)**
Sedentary proportion, %/day	71.0 (7.3)***	65.4 (8.1)***
**Physical activity**		
Accelerometry, days	24 (5)	26 (4)
Wear time, h/day	14.3 (1.1)	14.4 (1.0)
Breaks in sedentary, time/day	26 (7)**	30 (8)**
Standing, h/day	1.4 (0.4)***	2.2 (0.8)***
Standing proportion, %/day	10.1 (2.9)***	15.0 (5.0)***
Daily steps	5408 (2288)	5206 (2046)
LPA, h/day	1.7 (0.6)	1.8 (0.5)
LPA proportion, %/day	11.7 (3.9)	12.8 (3.1)
MVPA, h/day	1.0 (0.4)	0.98 (0.4)
MVPA proportion, %/day	7.3 (2.9)	6.8 (2.5)
PA, h/day	2.7 (0.9)	2.8 (0.7)
PA proportion, %/day	19.0 (5.8)	19.6 (4.8)

Significant p-values; *p < 0.05, **p < 0.01, ***p < 0.001. Sex difference in t-test (or Fisher’s exact test, when applicable).

ALT, alanine aminotransferase; AST, aspartate aminotransferase; GGT, γ-glutamyltransferase; LPA, light physical activity; MVPA, moderate to vigorous physical activity; PA, physical activity (LPA and MVPA together).

Male participants had statistically significant higher daily lying time, sedentary time, and sedentary proportion. Women had more breaks in daily sedentary time and their standing time and standing proportion were higher when compared to men. There were no differences between sexes in sitting time, daily steps, LPA, LPA (%), MVPA, MVPA (%), PA and PA (%). When comparing the health measurements men had statistically significant higher alcohol consumption and higher ALT, GGT, fasting insulin and HOMA-IR levels, and women had higher cholesterol and HDL levels. There were no significant differences between sexes in AST, systolic blood pressure (SBP), diastolic blood pressure (DBP), resting HR, fasting glucose, triglycerides, LDL, and HbA_1c._

### Correlations of Body Adiposity With PA and SB

BMI associated negatively with MVPA (r= -0.23, p=0.005), daily steps (r=-0.30, p<0.0001) and breaks in sedentary time (r=-0.32, p<0.0001). BMI associated positively with SB % (r=0.21, p=0.008) but not with total sedentary time (r=-0.04, p=0.62). WC associated with breaks in SB (r=-0.35, p<0.0001), MVPA (r=-0.26, p=0.002), daily steps (r=-0.31, p<0.0001), SB % (r=0.27, p=0.001) and standing time (r=-0.27, p=0.001), whereas with LPA there was no association (r=-0.13, p=0.13). All correlations were adjusted for age and sex.

### Correlations of ALT, AST and GGT With Body Adiposity

When adjusted with age and sex, ALT (r = 0.34, p < 0.0001; r = 0.41, p < 0.0001), AST (r = 0.17, p = 0.049; r = 0.26, p = 0.002) and GGT (r = 0.29, p = 0.0005; r = 0.32, p < 0.0001), were all positively associated with BMI and WC, respectively. Additionally, these enzymes were positively associated with SBP (ALT r = 0.27, p = 0.001; AST r = 0.18, p = 0.04; GGT r = 0.25, p = 0.004), DBP (ALT r = 0.27, p = 0.001; AST r = 0.23, p= 0.006; GGT r = 0.27, p = 0.001), resting HR (ALT r = 0.20, p = 0.02; AST r = 0.27, p = 0.002; GGT r = 0.25, p = 0.003), fasting insulin (ALT r = 0.48, p < 0.0001; AST r = 0.35, p < 0.0001; GGT; r = 0.31, p = 0.0002), HOMA-IR (ALT r = 0.46, p < 0.0001; AST r = 0.30, p = 0.0004; GGT r = 0.31, p = 0.0002) and HDL (ALT r = -0.24, p = 0.005; AST r = -0.19, p = 0.03; GGT r = -0.18, p = 0.04). Additionally, ALT (r = 0.30, p = 0.0004; r = 0.23, p = 0.007) and GGT (r = 0.18, p = 0.04; r = 0.22, p = 0.01) was positively associated with fasting glucose and triglycerides, respectively. Also, ALT (r = 0.30, p = 0.0004) and AST (r = 0.20, p = 0.02) was positively associated with HbA_1c_. However, ALT (r = -0.02, p = 0.77; r = -0.04, p = 0.68), AST (r = 0.04, p = 0.66; r = -0.04, p = 0.62) and GGT (r = 0.04, p = 0.67; r = 0.04, p = 0.63) had no statistically significant association with LDL or total cholesterol, respectively (**Table 2**).

**Table 2:** Age and sex-adjusted Pearson partial correlation coefficients between circulating liver enzymes (ALT, AST, GGT), anthropometrics and health measurements.

	ALT^a^, _U/l_	AST^a^, _U/l_	GGT^a^, _U/l_
**Anthropometrics**			
BMI, kg/m^2^	0.34***	0.17*	0.29***
Waist, cm	0.41***	0.26**	0.32***
**Health measurements**			
Systolic blood pressure, mmHg	0.27**	0.18*	0.25*
Diastolic blood pressure, mmHg	0.27**	0.23**	0.27**
Resting heart rate, bpm	0.20*	0.27**	0.25**
f-Glucose, mmol/l	0.30***	0.16	0.18*
f-Insulin, mU/l	0.48***	0.35***	0.31***
HOMA-IR^a^	0.46***	0.30***	0.31***
Triglycerides, mmol/l	0.23**	0.08	0.22**
Cholesterol, mmol/l	-0.04	-0.04	0.04
HDL, mmol/l	-0.24**	-0.19*	-0.18*
LDL, mmol/l	-0.02	0.04	0.04
HbA_1c_, mmol/mol	0.30***	0.20*	0.09

Significant p-values; * < 0.05, ** < 0.01, *** < 0.001

ALT, alanine aminotransferase; AST, aspartate aminotransferase; GGT, γ-glutamyltransferase; HOMA-IR, homeostatic model assessment for insulin resistance. ^a^ = log10 transformed variables.

### Correlations of ALT, AST and GGT With SB and PA

There were no statistically significant associations between circulating liver enzymes (ALT, AST and GGT) and aspects of SB; lying time (ALT r = -0.06, p = 0.45; AST r = -0.06, p = 0.52; GGT r = -0.03, p = 0.69), sitting time (ALT r = 0.05, p = 0.59; AST r = 0.03, p = 0.76; GGT; r = -0.03, p = 0.70), sedentary time (ALT r = 0.002, p = 0.98; AST; r = -0.01, p = 0.90; GGT r = -0.05, p = 0.54), SB % (ALT r = 0.04, p = 0.66; AST r = -0.008, p = 0.93; GGT; r = 0.03, p = 0.70). Additionally, we did not find any statistically significant associations between the liver enzymes and PA; standing time (ALT r = -0.08, p = 0.38; AST r = -0.06, p =0.49; GGT r = -0.12, p = 0.16), standing % (ALT r = -0.07, p = 0.41; AST r = -0.07, p = 0.44; GGT r = -0.11, p = 0.22), LPA (ALT r = 0.07, p = 0.45; AST r = 0.10, p = 0.24; GGT r = 0.04, p = 0.67), LPA% (ALT r = 0.09, p = 0.31; AST r = 0.11, p = 0.20; GGT r = 0.06, p = 0.46), MVPA (ALT r = -0.13, p = 0.14; AST r = -0.01, p = 0.89; GGT r = -0.02, p = 0.79), MVPA% (ALT r = -0.11, p = 0.18; AST r = -0.009, p = 0.91; GGT r = -0.005, p = 0.96), total PA (LPA and MVPA together) (ALT r = 0.02, p = 0.86; AST r = 0.14, p = 0.09; GGT r = 0.16, p = 0.06) total PA% (ALT r = 0.003, p = 0.97; AST r = 0.15, p = 0.09; GGT r = 0.16, p = 0.06), breaks in sedentary time (ALT r = -0.14, p = 0.10; AST r = -0.07; p = 0.44; GGT r = -0.17, p = 0.06), daily steps (ALT r = -0.15, p = 0.07; AST r = -0.03, p = 0.75; GGT r = -0.07, p = 0.44), when adjusted for age and sex (**Table 3**).

**Table 3:** Age and sex-adjusted Pearson partial correlation coefficients between circulating liver enzymes (ALT, AST, GGT), sedentary behavior and physical activity.

	ALT^a^, _U/l_	AST^a^, _U/l_	GGT^a^, _U/l_
**Sedentary behavior**			
Lying time, h/day	-0.06	-0.06	-0.03
Sitting time, h/day	0.05	0.03	-0.03
Sedentary time, h/day	0.002	-0.01	-0.05
Sedentary proportion, %/day	0.04	-0.008	0.03
**Physical activity**			
Breaks in sedentary, times/day	-0.14	-0.07	-0.16^# p=0.053^
Standing, h/day	-0.08	-0.06	-0.12
Standing proportion, %/day	-0.07	-0.07	-0.11
Steps, number/day	-0.15	-0.03	-0.07
LPA, h/day	0.07	0.1	0.04
LPA proportion, %/day	0.09	0.11	0.06
MVPA, h/day	-0.13	-0.01	-0.02
MVPA proportion, %/day	-0.11	-0.009	-0.005
PA, h/day	0.02	0.14	0.16
PA proportion, %/day	0.001	0.14	0.16

ALT, alanine aminotransferase; AST, aspartate aminotransferase; GGT, γ-glutamyltransferase; LPA, light physical activity; MVPA, moderate-to-vigorous physical activity; PA, physical activity (LPA and MVPA together). ^a^ = log10 transformed variables.

### Associations Based on Multivariable Models

Majority of the associations between liver enzymes and health variables observed in the sex and age adjusted correlation analysis were remained significant when BMI was added to the multivariable model. ALT was positively associated with WC (β = 0.41, 95% CI [0.002 – 0.01], p = 0.008), SBP (β = 0.20, 95% CI [0.0004 – 0.003], p = 0.01), DBP (β = 0.22, 95% CI [0.001 – 0.006], p = 0.005), resting HR (β = 0.17, 95% CI [0.0003 – 0.006] p = 0.03), fasting glucose (β = 0.19, 95% CI [0.006 – 0.08], p = 0.03), fasting insulin (β = 0.41, 95% CI [0.005 – 0.01], p < 0.0001), HOMA-IR (β = 0.42, 95% CI [0.02 – 0.04], p < 0.0001) and HbA_1c_ (β = 0.23, 95% CI [0.003 – 0.01], p = 0.003). However, the associations between ALT and triglycerides (β = 0.15, 95% CI [-0.003 – 0.07], p = 0.07) and HDL (β = -0.16, 95% CI [-0.15 – 0.008], p = 0.08) turned non-significant. AST was associated with WC (β = 0.37, 95% CI [0.0005 – 0.007], p = 0.03), DBP (β = 0.23, 95% CI [0.0007 – 0.004], p = 0.006), resting HR (β = 0.25, 95% CI [0.0009 – 0.004], p = 0.002), fasting insulin (β = 0.36, 95% CI [0.002 – 0.007], p = 0.0003), HOMA-IR (β = 0.36, 95% CI [0.007 – 0.02], p = 0.0003) and HbA_1c_ (β = 0.17, 95% CI [0.00008 – 0.006], p = 0.04). However, the association between SBP (β = 0.14, 95% CI [- 0.0001 – 0.002], p = 0.09) and HDL (β = - 0.15, 95% CI [- 0.09 – 0.01], p = 0.12) turned non-significant. GGT was associated with SBP (β = 0.19, 95% CI [0.0004 – 0.005], p = 0.02), DBP (β = 0.23, 95% CI [0.002 – 0.009], p = 0.004), resting HR (β = 0.23, 95% CI [0.002 – 0.01], p = 0.004), fasting insulin (β = 0.20, 95% CI [0.0004 – 0.01], p = 0.04) and HOMA-IR (β = 0.21, 95% CI [0.002 – 0.04], p = 0.03). However, the associations between GGT and WC (β = 0.22, 95% CI [-0.002 – 0.01], p = 0.16), fasting glucose (β = 0.08, 95% CI [-0.03 – 0.08], p = 0.37), triglycerides (β = 0.15, 95% CI [-0.004 – 0.10], p = 0.07) and HDL (β = -0.10, 95% CI [-0.18 – 0.05], p = 0.26) turned non-significant (**Table 4**). The associations between SB and PA measures with liver enzymes remained non-significant when BMI was added to the model (**Table 5**).

**Table 4:** Age, sex and BMI-adjusted linear mixed regression estimates (standardized β coefficients) between circulating liver enzymes (ALT, AST, GGT), anthropometrics and cardiometabolic risk factors.

	ALT^a^_(U/l)_	AST^a^_(U/l)_	GGT^a^_(U/l)_
	β	β	β
**Anthropometrics**			
Waist, cm	0.42**	0.37*	0.22
**Cardiometabolic biomarkers**			
Systolic blood pressure, mmHg	0.20*	0.14	0.19*
Diastolic blood pressure, mmHg	0.22**	0.23**	0.23**
Heart rate, bpm	0.17*	0.25**	0.23**
Blood pressure medication	-0.07	-0.05	-0.07
Cholesterol medication	-0.10	-0.001	-0.01
Glucose, mmol/l	0.19*	0.10	0.08
Insulin, mU/l	0.41***	0.36***	0.20*
HOMA-IR	0.42***	0.36***	0.21*
Triglycerides, mmol/l	0.15	0.04	0.15
Cholesterol, mmol/l	0.003	-0.007	0.08
HDL, mmol/l	-0.16	-0.15	-0.10
LDL, mmol/l	0.001	0.05	0.06
HbA_1c_, mmol/mol	0.23**	0.17*	0.03

Significant p-values; * < 0.05, ** < 0.01, *** < 0.001

ALT, alanine aminotransferase; AST, aspartate aminotransferase; GGT, γ-glutamyltransferase; HOMA-IR, homeostatic model assessment for insulin resistance. ^a^ = log10 transformed variables.

**Table 5:** Age, sex and BMI-adjusted linear mixed regression estimates (standardized β coefficients) between circulating liver enzymes (ALT, AST, GGT) and sedentary behavior and physical activity.

	ALT^a^, _U/l_	AST^a^, _U/l_	GGT^a^, _U/l_
	β	β	β
**Sedentary behavior**			
Lying time, h/day	-0.12	-0.09	-0.09
Sitting time, h/day	0.03	0.01	-0.05
Sedentary time, h/day	-0.05	-0.04	-0.10
Sedentary proportion, %/day	-0.05	-0.07	-0.09
**Physical activity**			
Breaks in sedentary, times/day	-0.02	-0.005	-0.06
Standing, h/day	0.02	0.02	-0.03
Standing proportion, %/day	0.02	0.0002	-0.02
Steps, number/day	-0.06	0.03	0.03
LPA, h/day	0.10	0.13	0.07
LPA proportion, %/day	0.11	0.13	0.09
MVPA, h/day	-0.05	0.03	0.05
MVPA proportion, %/day	-0.05	0.02	0.06
PA, h/day	0.008	0.14	0.15
PA proportion, %/day	-0.002	0.14	0.15

ALT, alanine aminotransferase; AST, aspartate aminotransferase; GGT, γ-glutamyltransferase; LPA, light physical activity; MVPA, moderate-to-vigorous physical activity. ^a^ = log10 transformed variables.

We also tested the associations between liver enzymes (ALT, AST and GGT) and the medications that had been used. We did not any association between the liver enzymes and blood pressure medication (ALT β = -0.07, 95% CI [-0.04 - 0.02], p = 0.42; AST β = -0.05, 95% CI [-0.02 – 0.01], p = 0.57; GGT β = -0.07, 95% CI [-0.06 – 0.03], p = 0.42) or cholesterol medication (ALT β = -0.10, 95% CI [-0.07 – 0.02], p = 0.22, AST β = -0.001, 95% CI [-0.03 – 0.03], p = 0.99; GGT β = -0.01, 955 CI [-0.07 – 0.06], p = 0.87), when adjusted with sex, age and BMI (**Table 4**). Additionally, we tested the associations between liver enzymes and other medications that had been used (hormonal medication, thyroid medication, antidepressants, gastrointestinal medication, pain medication, rheumatoid or osteoarthritis medication, allergy and asthma medication, medication for urinary problems, anticoagulants, sleep medication, medication for vision and hearing related issues, migraine medication, medication for restless legs syndrome, psoriasis medication and epilepsy medication). However, we did not find any association between liver enzymes and the used medications, when adjusted with sex, age and BMI (data not shown).

Additionally, we ran the statistical models including the interaction of gender and physical activity measurements as well as interaction between age and physical activity measurements. Association between ALT and total PA, as well as PA % was statistically significantly different between males and females so that negative association was noticed for males and positive association for females. In GGT analyses it was noticed that association between GGT and MVPA, MVPA % and steps was different across age so that negative association was noticed for individuals who were < 50 years old and positive associated with individuals who were ≥ 50 years old. In all other variables, no significant interactions were noticed (data not shown).

We also ran the analysis with accelerometer wear time/day and valid accelerometer days included in the statistical model to adjust for differences in wear time and accelerometer days. When wear time was included in the model, significant association between GGT and total PA (β = 0.15, 95% CI [0.001 – 0.11], p = 0.046) was observed. The same association was also found when accelerometer days were included in the model, GGT was positively associated with total PA (β = 0.16, 95% CI [0.001 – 0.11], p = 0.047). In all other variables, however, no significant interactions were noticed (data not shown).

We also tested the associations between liver enzymes (ALT, AST and GGT) and the reported alcohol consumption. We did not find any significant associations between ALT and AST and alcohol consumption, when adjusted for age, sex and BMI. Further we also tested if the positive association between GGT and health variables (SBP, DPB, resting HR, fasting insulin and HOMA-IR) would change if we added alcohol consumption in the model. However, all the associations between GGT and cardiometabolic markers remained significant when adjusted for age, sex, BMI and alcohol consumption (data not shown).

Lastly, we tested the associations between the AST/ALT ratio (log10 transformed) and SB, PA and the health markers, when adjusted for sex, age and BMI. We did not find any significant associations between AST/ALT ratio and SB and PA. AST/ALT ratio was negatively associated with SBP (β = -0.17, 95% CI [-0.002 – 0.0001], p = 0.03), fasting glucose (β = -0.19, 95% CI [-0.05 - -0.005], p = 0.02), fasting insulin (β = -0.30, 95% CI [-0.007 - -0.002], p = 0.001), HOMA-IR (β = - 0.32, 95% CI [-0.02 - -0.007], p = 0.001), triglycerides (β = -0.19, 95% CI [-0.05 - -0.005], p = 0.02) and HbA_1c_ (β = -0.20, 95% CI [-0.007 - -0.001], p = 0.01).

## Discussion

In the present study we show that circulating liver enzymes (ALT, AST and GGT) were not associated with accelerometer-measured SB or habitual PA in inactive middle-aged population with overweight or obesity. However, we found that these enzymes were strongly associated with body adiposity (high BMI and WC) and other health risk markers (SBP, DBP, insulin resistance and resting HR). Thus, increased levels of liver enzymes appear to cluster with other common cardiometabolic risk factors. To our knowledge, this is the first study to study the associations between specific serum liver enzymes (ALT, AST and GGT) and device-measured elements of SB and PA tracked for longer period (4 weeks) in working-age adults with overweight or obesity.

### Association of Liver Enzymes With Health Markers

In line with our hypothesis, we found that all liver enzymes investigated (ALT, AST and GGT) were positively and strongly associated with common markers for obesity (BMI and WC). Previous studies have shown similar results (4, 5, 22, 23). We also found that ALT, AST and GGT remained positively associated with other common cardiometabolic risk factors, such as increased blood pressure, fasting insulin, and HOMA-IR, when adjusted for age, sex and BMI. Same kind of results have been reported earlier with specific risk factors (24, 25). For instance, Rahman et al. (24) studied Bangladeshi adults in a cross-sectional setting and found that increased serum ALT and GGT levels were both positively and independently associated with hypertension in both men and women. Marchesini et al. (25) found that ALT, AST and GGT were all positively associated with HOMA-IR in adults with obesity (BMI >30), when adjusted for age, sex and BMI. Additionally, ALT (but not AST or GGT) was positively associated with fasting glucose and HbA_1c_. It has also been shown that rather high ALT levels, but not AST or GGT are associated with metabolic diseases like type II diabetes (26). As we did not find any associations between these three liver enzymes and triglycerides, LDL and total cholesterol, our results suggest that liver enzymes are more strongly associated with factors related to plasma glucose profile rather than lipid profile.

An interesting observation in our study was that resting HR correlated positively with all three liver enzymes and the correlation remained significant with further adjustment for BMI. Results were also reported in a study by Straznicky et al. (27), in which both ALT and GGT were positively associated with resting HR in obese subjects with metabolic syndrome. Additionally, Kim et al. (28) recently reported a positive association between resting HR and non-alcoholic fatty liver disease (NAFLD) in post-menopausal women. Associations of resting HR and blood pressure with liver enzymes independent of obesity may reflect heightened stress levels and autonomic nervous system imbalance, clustered together with impaired glucose metabolism as observed also in the present study. Thus, our results add new insights to the existing evidence, suggesting that measuring resting HR might be an easy additional way to assess the risk of liver diseases in people with overweight. However, we did not include other cardiometabolic risk factors (such as high blood pressure, high fasting insulin levels) in the model which could also explain the association between liver enzymes and resting HR. Therefore, future studies should aim to verify these assumptions.

### Associations Between Liver Enzymes and SB and PA

We hypothesized that liver enzymes would show a positive association with sedentary time and a negative association with standing and breaks in sedentary time after adjustments for age, sex and especially after adjusting for BMI. In contrast to our hypothesis, we did not find any associations between liver enzymes and SB, nor standing or breaks in SB. Previously, Mor et al. (29) found that lack of regular PA is associated with increased levels of ALT when adjusted for central obesity, alcohol consumption, and comorbidity in newly diagnosed adult type II diabetes patients. However, the study was based on self-reported exercise frequency (days per week) and it has been shown earlier that there are various limitations related to self-reported methods (30), such as over-reporting the amount and intensity of PA (31). However, Li et al. (18) found that accelerometer-measured sedentary time was independently associated with increased ALT and GGT levels after adjustment for basic cardiometabolic markers (such as BMI) in US Hispanic/Latino adults. Difference in results between our study and Li et al. study may be due to difference in study populations used, as their study included participants of different ethnicity than ours, and they also used a wider age range. On the other hand, there are also studies that are in line with our results, in which accelerometer-measured sedentary time is not associated with circulating liver enzymes (ALT and AST) (16, 17).

We further hypothesized that liver enzymes (ALT, AST and GGT) would show negative association between LPA and MVPA. However, we did not find any association between these variables or with any other PA measures when adjusted for age, sex, and BMI. A similar conclusion concerning ALT was reached by Hallsworth et al. (32) who did not find any correlation between ALT and objectively measured sedentary time or PA parameters in subjects diagnosed with NAFLD. Also, Bacchi et al. (33) showed that neither ALT, AST nor GGT were associated with aerobic training or resistance training in subjects with type II diabetes and NAFLD.

However, mixed results also regarding the effects of exercise training on liver enzymes have been reported. Fragala et al. (34) found in adults aged 18-34 years that more days of aerobic or resistance training was associated with higher levels of AST in men and lower levels of ALT and GGT in both men and women. Additionally, exercise training decreased ALT levels in people with obesity (35, 36). In contrast, Petterson et al. (37) found that acute resistance training session increased AST and ALT levels in heathy men for at least seven days after the exercise had been performed, suggesting that strenuous muscular exercise can maintain higher liver enzyme levels for a fairly long period of time. However, regular exercise training has potential to lower these enzyme levels (34–36).

In general, previous studies concerning the associations between liver enzymes and SB or PA have been conflicting and it appears that no concrete conclusions can be drawn due to mixed results. Differences might originate from the methods that were used to measure PA and SB and the different populations studied, but it appears that more formal exercise training rather than habitual PA or avoidance of sitting is needed to lower liver enzyme levels in obese subjects (35, 36). Further, aminotransferase levels rise in the blood when liver is injured but it has been shown that people with liver disease do not necessarily have abnormal levels of these enzymes (38). Thus, one reason for mixed results might be the genetic differences in the liver enzyme levels (39), and also the differences in the levels of physical fitness (40). However, despite the differences between the results, there is convincing evidence that increased levels of liver enzymes are associated with liver disease (22, 41) and liver-related mortality (23) and thus circulating liver enzymes are commonly used in clinical practice as biomarkers of liver injury.

### Strengths and limitations

One strength of our study pertains to the use of accelerometer and validated algorithms in measuring SB and PA measures in contrast to less precise, self-reported methods that have been used in many previous investigations (29, 34, 42). Also, the relatively long measuring time (4-weeks) can be counted as strength when compared to other studies with shorter PA tracking times. Additionally, the subjects in our study used the accelerometer in a free-living environment which can account for a strength when compared to laboratory settings.

To assess PA and SB we used validated hip-worn accelerometers. It has been reported that hip-worn accelerometers are more accurate in assessing PA and estimating energy consumption when compared to wrist-worn meters (43). However, all methods that measure PA have always some limitations; most accelerometers are not able to detect the true intensity of some activities (such as swimming, resistance training or carrying heavy loads) and therefore the overall strenuousness of PA might have been underestimated. Also, more detailed information about the association between resting HR and liver enzymes and the diet, part from alcohol intake, would have provided more insights to our results, but it was beyond the scope of this study and can thus be considered a limitation.

One factor in this study that could have affected the results was that we use only the inactive participants. It is possible that if our participants would have met the PA recommendations the results might have been different. However, our results could refer that the intensity of PA can play important role, and light habitual PA (such as standing, breaks in sitting, LPA) might not be enough stimuli to have a positive effect on the liver enzymes.

The other limitations of the present study include that the cut-off point for separating sitting from lying was not previously validated and the cross-sectional setting which prevents the causal interpretation of these results. Therefore, future studies should aim to assess the relationship between liver enzymes and habitual PA and SB in longitudinal and experimental settings, which may show causal relations.

## Conclusions

Objectively measured SB (sitting, lying) or PA (breaks in SB, steps, standing, LPA, MVPA, total PA) were not associated with liver enzymes (ALT, AST and GGT) in the present study in inactive subjects with overweight and obesity. However, body adiposity (BMI, WC) was strongly associated with liver enzymes. These results suggest that different elements of SB or PA are not essential in modulating the levels of circulating liver enzymes if the current guidelines for PA are not met. Achieving and maintaining healthy body adiposity may be more important than habitual PA per se, although SB and PA markers are associated with obesity markers as observed also in the present study, indicating that they also have potential to contribute to obtaining healthy body adiposity.

## Data Availability Statement

The original contributions presented in the study are included in the article/supplementary material. Further inquiries can be directed to the corresponding author.

## Ethics Statement

The studies involving human participants were reviewed and approved by The Ethics Committee of the Hospital District of Southwestern Finland (Turku, Finland, study permission no TO5/026/17). The patients/participants provided their written informed consent to participate in this study.

## Author Contributions

IHA, JK, TV, and TS conception and design of the study. SL, TS, and TG, acquisition of data. SL, TS, HV-Y, TG, EL, HS, and IHA analysis and interpretation of data. SL drafted the manuscript and all authors edited and revised the manuscript. All authors contributed to the article and approved the submitted version.

## Funding

The study was financially supported by the Academy of Finland (324243), Instrumentarium Science Foundation (200034), Turku UniversityFoundation (080519), Juho Vainio Foundation (202010203), Hospital District of South-West Finland (13282), Finnish Diabetes Research Foundation (180021), Finnish Cultural Foundation (181019) and Yrjö Jahnsson Foundation (20187112).

## Conflict of Interest

The authors declare that the research was conducted in the absence of any commercial or financial relationships that could be construed as a potential conflict of interest.

## Abbreviations

ALT, alanine aminotransferase; APE, angle for posture estimation; AST, aspartate aminotransferase; BMI, body mass index; DBP, diastolic blood pressure; GGT, γ-glutamyltransferase; HOMA-IR, homeostatic model assessment for insulin resistance; HR, heart rate; LPA, light physical activity; LPA (%), daily proportion of light physical activity; MAD, mean amplitude deviation; MET, metabolic equivalent; MVPA, moderate-to-vigorous physical activity; MVPA (%), daily proportion of moderate-to-vigorous physical activity; NAFLD, non-alcoholic fatty liver disease; PA, physical activity (LPA and MVPA together); PA (%), daily proportion of physical activity; SB, sedentary behavior; SBP, systolic blood pressure**;** WC, waist circumference.
